# Development of a Yoga-Based Cardiac Rehabilitation (Yoga-CaRe) Programme for Secondary Prevention of Myocardial Infarction

**DOI:** 10.1155/2019/7470184

**Published:** 2019-05-02

**Authors:** Kaushik Chattopadhyay, Ambalam M. Chandrasekaran, Pradeep A. Praveen, Subhash C. Manchanda, Kushal Madan, Vamadevan S. Ajay, Kavita Singh, Therese Tillin, Alun D. Hughes, Nishi Chaturvedi, Shah Ebrahim, Stuart Pocock, K. Srinath Reddy, Nikhil Tandon, Dorairaj Prabhakaran, Sanjay Kinra

**Affiliations:** ^1^The University of Nottingham, Nottingham, UK; ^2^London School of Hygiene and Tropical Medicine, London, UK; ^3^Centre for Chronic Disease Control, New Delhi, India; ^4^All India Institute of Medical Sciences, New Delhi, India; ^5^Sir Ganga Ram Hospital, New Delhi, India; ^6^Public Health Foundation of India, Gurgaon, India; ^7^University College London, London, UK; ^8^Imperial College London, London, UK

## Abstract

Cardiac rehabilitation (CR) after myocardial infarction is highly effective. It is unavailable in public hospitals in India due to limited resources. Our objective was to develop a scalable model of CR for India based on yoga, which could also appeal to some groups with low uptake of CR (e.g., ethnic minorities, women, and older people) globally. The intervention was developed using a structured process. A literature review and consultations with yoga experts, CR experts, and postmyocardial infarction patients were conducted to systematically identify and shortlist appropriate yoga exercises and postures, breathing exercises, meditation and relaxation practices, and lifestyle changes, which were incorporated into a conventional CR framework. The draft intervention was further refined based on the feedback from an internal stakeholder group and an external panel of international experts, before being piloted with yoga instructors and patients with myocardial infarction. A four-phase yoga-based CR (Yoga-CaRe) programme was developed for delivery by a single yoga instructor with basic training. The programme consists of a total of 13 instructor-led sessions (2 individual and 11 group) over a 3-month period. Group sessions include guided practice of yoga exercises and postures, breathing exercises, and meditation and relaxation practices, and support for the lifestyle change and coping through a moderated discussion. Patients are encouraged to self-practice daily at home and continue long-term with the help of a booklet and digital video disc (DVD). Family members/carers are encouraged to join throughout. In conclusion, a novel yoga-based CR programme has been developed, which promises to provide a scalable CR solution for India and an alternative choice for CR globally. It is currently being evaluated in a large multicentre randomised controlled trial across India.

## 1. Introduction

**Figure 1:**
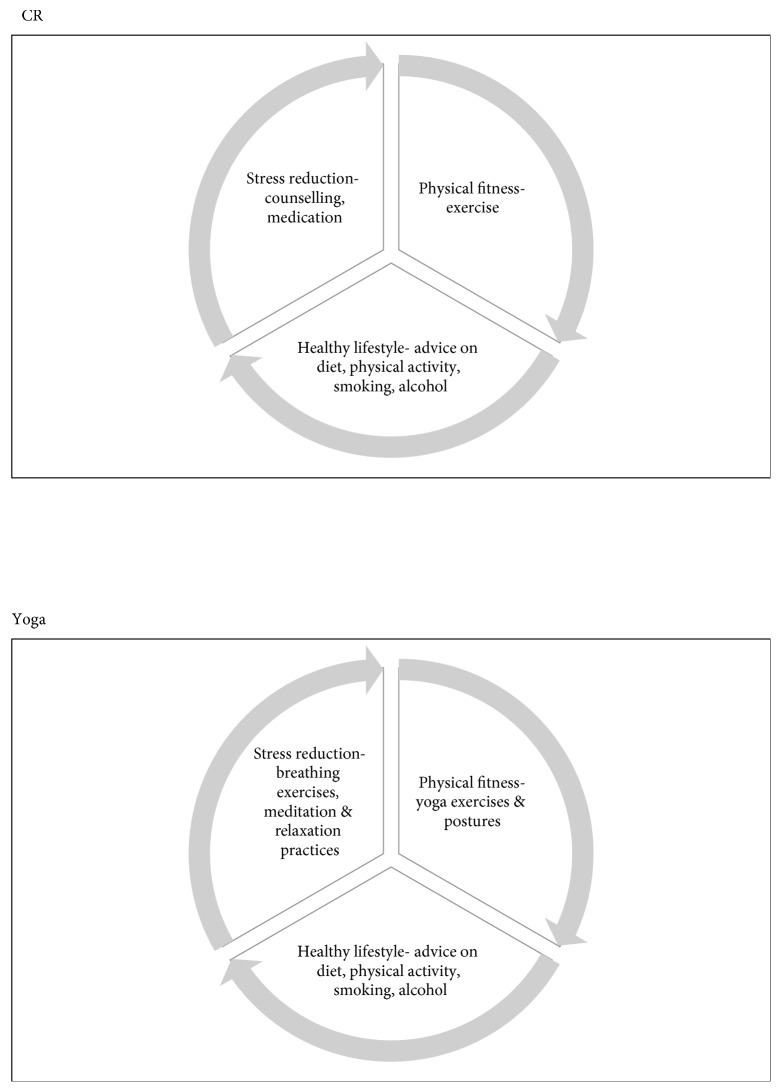
Similarities between CR and yoga.

Secondary prevention of myocardial infarction reduces associated morbidity and mortality [1]. One approach is cardiac rehabilitation (CR), a programme of information and exercise sessions, which aims to help patients recover from myocardial infarction and reduce lifestyle risk factors for cardiovascular disease [1]. CR programmes have been shown to reduce morbidity and mortality, improve quality of life, lower medical and social costs, and increase economic productivity [2–4]. As a result, CR programmes are recommended as the standard by major clinical guidelines [5]. Despite this, the uptake of CR programmes remains low globally [6, 7]. CR requires large multidisciplinary teams, which are not widely available in low- and middle-income countries (LMICs), such as in India, especially in public hospitals with limited resources [6–10]. In high-income countries (HICs), CR programmes are widely available, but participation is still poor, especially among population subgroups, such as ethnic minorities, women, and older people who may find the vigorous forms of exercise and communication style of established CR programmes unappealing [3, 6, 7]. Alternative models of CR based on traditional eastern practices (e.g., yoga and tai chi), which have increasing global acceptance despite their distinct approaches, may offer scalable solutions for LMICs and greater choice in HICs [11]. Yoga is an ancient Indian mind-body discipline [12]. Of the seven major branches of yoga, hatha yoga is probably the most commonly recognised [13]. It aims to build physical and mental strength through yoga exercises and postures, breathing exercises, meditation and relaxation practices (which also reduces stress), and moderation in lifestyle [13]. With its emphasis on physical fitness, stress reduction, and healthy lifestyle, yoga covers most of the elements of a conventional CR programme (see Figure 1) and yet could be delivered by a single yoga instructor with minimal training. The aim of this study was to systematically develop a yoga-based CR (Yoga-CaRe) programme for the secondary prevention of myocardial infarction. The intention was to develop a low-cost scalable programme that could be easily integrated within the existing care (with the help of a single yoga instructor with basic training) rather than requiring additional resources for activities (e.g., diagnostics) that are not part of the existing care (and would limit scalability).

## 2. Materials and Methods

The Yoga-CaRe programme was developed through a systematic process based on the UK's Medical Research Council (MRC) guidance on developing and evaluating complex interventions and Sherman's guidelines for developing yoga interventions for randomised controlled trials [14, 15]. As part of the intervention, a manual for instructors and a booklet and a high-definition digital video disc (DVD) for participants were developed. All these are available in English and in major Indian languages. An internal stakeholder group and an external panel of international experts (details below) guided its development. The study was approved by the research ethics committees of the London School of Hygiene and Tropical Medicine (UK) and Centre for Chronic Disease Control (India).

The development process consisted of seven steps. First, a CR framework was developed based on a review of major established CR guidelines [16–19]. In this review, the key features of CR programmes were identified.

Second, a literature review and interviews with experts were conducted to identify the yoga exercises and postures, breathing exercises, meditation and relaxation practices, and lifestyle changes recommended for patients with heart disease in the yoga system. We hand searched publications from the yoga institutes of the Government of India, Moraji Desai National Institute of Yoga and Central Council for Research in Yoga and Naturopathy, as they are not easily searchable online. Face-to-face qualitative interviews were conducted with six yoga experts purposively selected to represent major yoga schools of thought, expertise (philosophy, research, or practice), and gender. A semistructured interview schedule was used, and interviews were tape-recorded and transcribed verbatim.

Third, the key yoga exercises and postures, breathing exercises, meditation and relaxation practices, and lifestyle changes were prioritised for inclusion in the Yoga-CaRe programme through a questionnaire survey of the stakeholder group (Intervention Development Steering Group), which included (a) seven CR experts representing a typical CR multidisciplinary team: cardiologist, physiotherapist, exercise physiologist, occupational therapist, psychologist, specialist cardiac nurse, and dietician (only for the diet component); (b) four postmyocardial infarction patients who practised yoga after myocardial infarction representing two age groups (< or ≥ 60 years) and gender. The questionnaire was administered electronically via email. It asked the respondents to rate the identified yoga exercises and postures, breathing exercises, meditation and relaxation practices, and lifestyle changes (identified in the previous step) for safety (by CR experts) and acceptability (by postmyocardial infarction patients) on a four-point Likert scale (strongly agree, somewhat agree, somewhat disagree, and strongly disagree). The items rated as “strongly agree” and “somewhat agree” by > 50% of the participants were shortlisted as core and elective items for the CR programme, respectively.

Fourth, the proposed intervention, the manual for instructors and the booklet for participants, was drafted by incorporating the agreed core and elective items from the previous step into the CR framework (from the first step).

Fifth, the proposed intervention was discussed at a face-to-face workshop of the Intervention Development Steering Group in India. The group deliberated on the overall structure and functionality of the proposed intervention, which led to further refinement of the intervention.

In the sixth step, the proposed intervention was shared with the International External Advisory Panel, which consisted of eight UK and Indian experts with either a scientific or practical background in CR or yoga. These external members further reviewed the overall structure and functionality of the proposed intervention.

In the final step, the proposed intervention was piloted and finalised. The piloting was conducted in India among (a) four yoga instructors (representing two levels of professional experience (< or ≥ 10 years' experience) and gender); (b) four postmyocardial infarction patients (representing two age groups (< or ≥ 60 years) and gender). Feedback was sought on the acceptability of the intervention, overall sequence, and flow of the intervention, timing, and comprehension of content/instructions.

After finalising the booklet for participants, it was also converted into a high-definition DVD to aid audio-visual learning. For this purpose, an external filmmaking company was hired in India.

## 3. Results

The Yoga-CaRe programme has four phases (see Table 1). It is comprised of 13 sessions delivered by a single yoga instructor with basic training, over a period of 3 months, with encouragement to self-practice daily. While the programme is primarily directed towards patients with myocardial infarction, their family members/carers are also encouraged to join where possible. The programme is primarily delivered in the hospital premises by the instructor, and patients are under the medical care and supervision of a cardiologist. The instructors are trained regularly by a team of cardiologists, CR experts, and yoga experts. The training topics include the Yoga-CaRe programme, anatomy and physiology of the cardiovascular system, communicating with patients, barriers and facilitators to CR, and identifying warning signs and distress symptoms.

Phase I involves an individual session with the patient while they are still in the hospital following admission for myocardial infarction. It is primarily an educational session aimed at providing information about the health condition and its treatment, recovery process, and practical advice about the lifestyle and activities of daily living. Topics like healthy diet (e.g., eating plenty of fruit and vegetables, cutting down on salt, sugar, saturated fats, and trans fats), physical activity, alcohol consumption, smoking/tobacco cessation, healthy weight and body shape, mental stress, medicines intake, high blood pressure, high blood cholesterol, diabetes, driving, returning to work, and sex are covered.

Phase II involves an individual session during the third week after myocardial infarction. The timing of this session coincides with the patient's first outpatient review after myocardial infarction with their cardiologist. During this session, the patient is introduced to the yoga element of the programme (i.e., breathing exercises and meditation and relaxation practices) and taught how to practice these correctly (see Table 2).

Phase III lasts from 5th to 12th week after myocardial infarction. During this period, the patient is invited to attend group sessions. While they are encouraged to attend as often as possible, they are strongly encouraged to attend at least twice a week during the first three weeks (5th to 7th week after myocardial infarction) and once a week for the following five weeks (8th to 12th week after myocardial infarction). The sessions typically last for an hour and 15 minutes (see Table 3). In each session, three warm-up exercises are followed by a series of yoga exercises and postures, breathing exercises, and meditation and relaxation practices. To allow some variability in the routine and an element of personalisation based on personal fitness, multiple options are provided for each of the categories of practice, from which the instructor is asked to choose two core and one elective practices at each session. Towards the end of each session, patients spend time discussing issues and sharing experiences (moderated by the instructor) related to these practices, making lifestyle changes, and coping (physically, emotionally, and socially) with activities of daily living as they return to previous activities and relationships. During this time, the patient is also encouraged to start self-practice on other days of the week, slowly increasing it to daily practice by the end of this period.

Phase IV involves the long-term maintenance of lifestyle changes and self-practice of yoga exercises and postures, breathing exercises, and meditation and relaxation practices at home on most days using the booklet and DVD provided.

## 4. Discussion

We report the systematic development of a structured yoga-based CR programme. It is currently being evaluated in a major randomised controlled trial across India. 3,959 patients aged 18–80 years with acute myocardial infarction are recruited from 24 cardiac centres in India. Participants are followed 3-monthly until the end of the study. The co-primary outcomes are (a) time to occurrence of the first cardiovascular event (a composite of all-cause mortality, nonfatal myocardial infarction, nonfatal stroke, and hospitalisation for emergency revascularisation, unstable angina, and heart failure) and (b) quality of life at 12 weeks. The secondary outcomes are the need for any revascularisation procedure (coronary artery bypass surgery (CABG) or percutaneous coronary interventions (PCI)), return to preinfarct daily activities, tobacco cessation, adherence to prescribed medications, cognitive function, anxiety and depression, and cost-effectiveness of the programme [20]. If found to be effective and/or cost-effective, it will offer a scalable solution for the provision of CR in India and a potentially appealing CR option for certain disadvantaged groups globally. It may also serve as a template for greater use of other local (mind-body) practices for the scalable and culturally appropriate provision of CR globally.

The Yoga-CaRe programme offers several potential advantages. It can be delivered by a single yoga instructor with minimal training, instead of a large multidisciplinary team, making it eminently scalable in low-resource and remote settings. In high-resource settings, it may be more appealing to certain subgroups of patients (e.g., some ethnic minorities, women, and older people) who currently do not take part in CR because they do not feel comfortable with the more vigorous exercise forms and communication strategies used in the existing CR programmes. Yoga is already promoted widely by physical therapists as a safe and low-impact activity that can provide comparable benefits to well-designed exercise programmes, increasing general health and fitness, reducing stress, and variably improving a range of health conditions [21]. Furthermore, some studies show that yoga can have beneficial effects on blood pressure, glucose and lipid homeostasis, body weight, and functional capacity through a range of hormonal and neuroendocrine pathways [22]. Effects of yoga practice on subclinical cardiovascular measures, risk factors, and neuroendocrine pathways following acute coronary events are investigated in a parallel mechanistic study to the main clinical trial [23].

The structure and low-cost of the Yoga-CaRe programme also makes it feasible to encourage family members/carers to attend, which could potentially reduce patient anxiety and improve long-term adherence to the programme. The Yoga-CaRe programme includes a mix of (a) supervised centre-based and unsupervised home-based sessions and (b) individual and group sessions. Although some studies have found unsupervised home-based programmes (e.g., the Heart Manual) to be as effective as supervised centre-based programmes [24], a completely self-supervised CR was deemed to be inappropriate for wider use in India due to low levels of literacy, particularly among those attending public hospitals. Similarly, a mix of individual and group sessions was favoured to cope with variable levels of fitness and comprehension and yet provide benefits of group sessions from shared experiences and peer support. We have also incorporated flexibility within a structured programme, to prevent boredom from the similarity of routine, but at the same time allow standardised delivery and evaluation.

The study has a number of strengths and weaknesses. This is one of the few studies to report the systematic development of a yoga-based intervention. We used a systematic process that not only combined evidence from a range of sources (including alternative forms of literature for yoga), but also helped to reach consensus on this complex intervention. This approach has been widely used in the development of other rehabilitation programmes [25]. The close involvement of CR experts and postmyocardial infarction patients in the intervention development ensured that issues of safety and acceptability were fully explored. Uniquely, an attempt was made to systematically integrate western and traditional medical systems in the development of this intervention, which at times proved challenging and occasionally contradictory (such as dietary advice); in such cases, the western medical system was prioritised to ensure integration in formal healthcare services. We expect that other similar attempts will follow suit and contribute to strengthening the methodologies for the development of such cross-cultural interventions.

## 5. Conclusions

A novel yoga-based CR programme—Yoga-CaRe—has been systematically developed that could provide a scalable CR solution for India and greater choice for CR globally. It is currently being evaluated in a large multicentre randomised controlled trial across India.

## Figures and Tables

**Table 1:** Four phases of the Yoga-CaRe programme.

Phase		Week after myocardial infarction	Type of care
I	Inpatient care	1st week (Session 1)	Face-to-face individual session- education

II	Formal outpatient session-I	3rd week (Session 2)	Face-to-face individual session- yoga (supervised)

III	Formal outpatient session-II	5th to 7th week (Sessions 3-8; twice/week) 8th to 12th week (Sessions 9-13; once/week)	Face-to-face group sessions- yoga (supervised) & education; during the rest of the week, self-practice of yoga at home using the booklet & DVD provided

IV	Long-term maintenance of lifestyle changes & self-practice of yoga at home	13th week & beyond	Maintenance of lifestyle changes & self-practice of yoga at home on most days using the booklet & DVD provided

**Table 2:** Components of the formal outpatient session-I.

	Items	Time
Breathing exercises	(1) Anulom vilom/nadishodhana pranayam (without kumbhak) (alternate nostril breathing) (2) Bhramari pranayama (bee breathing) (3) Ujjayi pranayam (loud breathing)	Around 15 minutes for 3- each one for about 5 minutes

Meditation & relaxation practices	(1) Chanting (2) Mindfulness meditation (3) Shavasana (relaxation training)	Around 15 minutes for 3- each one for about 5 minutes in a darkened room

**Table 3:** Components of the formal outpatient session-II.

	Core items	Elective items	Time
	(select 2 out of 3)*∗*	(select 1 out of 2)	
Health rejuvenating exercises	(1) Shoulder exercises (2) Chest exercises (3) Abdomen exercises		Around 9 minutes for 3- each one for about 3 minutes

Yoga poses- standing	(1) Katichakrasana (waist wheel pose) (2) Tadasana (palm tree pose) (3) Urdhvahastottanasana (up stretched arms pose)	(1) Ardha-katichakrasana (lateral arc pose) (2) Trikonasana (triangle pose)	
Yoga poses- sitting	(1) Gomukhasana (cow face pose) (2) Janushirsasana (head on the knee pose) (3) Vakrasana (twisted pose)	(1) Ardha-padmasana (half lotus pose) (2) Vajrasana (adamant pose)	Around 25 minutes for 9 (3 standing, 3 sitting, & 3 lying)- 2-sided poses for about 3 minutes (1.5 minutes on each side- right & left) & central-positioned poses for about 1.5 minutes
Yoga poses- lying	(1) Ekpadottanasana (half-leg raise pose) (2) Naukasana (boat pose) (3) Ardha-pavanamuktasana (wind releasing pose)	(1) Markatasana (monkey pose) (2) Merudandasana (spinal cord pose)	

Breathing exercises	(1) Anulom vilom/nadishodhana pranayam (without kumbhak) (alternate nostril breathing) (2) Bhramari pranayama (bee breathing) (3) Ujjayi pranayam (loud breathing)	(1) Sitali pranayam (tongue hissing) (2) Sitkari pranayam (teeth hissing)	Around 15 minutes for 3- each one for about 5 minutes

Meditation & relaxation practices	(1) Chanting (2) Mindfulness meditation (3) Shavasana (relaxation training)	(1) Dirghasvasa preksha (perception of deep breathing) (2) Antaranga trataka (internal concentrated gazing)	Around 15 minutes for 3- each one for about 5 minutes in a darkened room

Moderated discussion	(1) Lifestyle changes (2) Self-practice of yoga at home (3) Any life issues (common problems, issues, or crises)		Around 10 minutes

*∗*Health rejuvenating exercises & discussion- all the 3 core items are to be selected.

## Data Availability

The data used to support the findings of this study are available from the corresponding author upon request, unless there are legal or ethical reasons for not doing so.
